# Mental health and Chemsex: A view from the Liaison and Emergency Psychiatry Service

**DOI:** 10.1192/j.eurpsy.2025.1247

**Published:** 2025-08-26

**Authors:** P. Muñoz-Calero, M. Navas Tejedor, A. Fuentes Merlo, L. Nuñez Cantos, D. Gimeno Álvarez, P. De la Cruz Ballano, A. Ramírez Ibernon

**Affiliations:** 1 Instituto de Psiquiatría y Salud Mental, Hospital Clínico San Carlos, Madrid, Spain

## Abstract

**Introduction:**

Chemsex is typically described as the use of specific substances to enable or enhance sex during extended sessions with multiple partners among men who have sex with men (MSM). Numerous studies have linked chemsex with different physical and mental health risks and other potential harms.

According to the latest European online survey por MSM (EMIS-2017), the section on substance use revealed the prevalente of substance use for sexual purposes in the last twelve months was 14.1% with Spain being one of the countries with the highest prevalence of chemsex at the European level.

As the prevalence of chemsex is increasing, consequences from this phenomena arises and a progressive increase in the number of psychiatric consultations and admissions related to chemsex practice have also been described, with substance abuse disorders, depression, and anxiety as the most prevalent diagnoses.

From our Liaison and Emergency Psychiatry Service consultations related with mental health issues and chemsex are increasing with growing number of suicide attempts, psychotic, maniac and depressive episodes.

**Objectives:**

Our objective in this poster is to describe the mental health risks of chemsex, its peculiarities and the main manage issuses in the Liaison and Emergency Pscychiatry Service.

**Methods:**

Systematic review of the scientific literature related with chemsex and mental health risks will be carried out and we will also analyse some of our cases to describe presentation and management of the symptoms.

**Results:**

Chemsex is related with an increased rate of mental health issues and consultations related with substance use disorders, depression and anxiety. Clinical features due to this disorders are unique and integral treatment in coordination with Infectious Medicine Specialist is needed.

**Conclusions:**

Chemsex is an increasing phenomena and it is intimately related with psychiatric disorders.

Psychiatrists must be concerned about it and trained to approach and treat these complex disorders in an integrative way.

**Disclosure of Interest:**

None Declared

